# Presenting a model for estimating the cube compressive strength of self-compacting concrete in cast in-situ piles using GEP

**DOI:** 10.1038/s41598-024-75608-6

**Published:** 2024-10-28

**Authors:** Hossein Maleki Toulabi, Seyed Azim Hosseini

**Affiliations:** Department of Civil Engineering, South Tehran Branch, Islamic Azad University, Tehran, Iran

**Keywords:** Self-compacting concrete, GEP, ANN, In-situ concrete, Engineering, Civil engineering

## Abstract

The cast in-situ pile is a widely used type of deep foundations which its execution in civil projects is increasing daily. The use of ordinary concrete in this type of piles causes technical and executive problems, a decrease in the compressive strength (CS) of concrete, and an increase in the permeability under the ground level. But use of the self-compacting concrete in the cast in-situ piles while increasing the CS of concrete ensures proper compaction, increase in the execution speed, and easy placing of concrete. In this article, utilizing the data obtained from the laboratory results and also the application of soft computing techniques, predicting the degree of CS of self-compacting concrete (SCC) in concrete piles was investigated. To estimate the CS of SCC, a total number of 7 inputs were implemented. Then, using gene expression programming (GEP) a model was presented for estimating the CS of SCC in the cast in-situ piles. The results of the neural network showed a precision of 99.98% which exhibits the high accuracy of the model. The use of this model could greatly help persons, companies, and research centers in the preparation and construction of self-compacting concrete with the desired CS.

## Introduction

The use of concrete cast in-situ deep foundations is continuously increasing. Application of this type of foundation in such projects as bridge piers, high-rise buildings, marine structures, and heavy industrial structures is of special importance. Foundations have a major share in maintaining the gravity stability and side stability of structures and the safety of important structures. Therefore the importance of proper transfer of heavy loads by executed piles is revealed. Alongside the considerable advantages of cast in-situ piles in terms of load bearing, the main shortcoming belongs to their concrete placement. After drilling, the issues that might affect the proper placement of concrete include a decrease in the tremie pipe diameter and damage to its section or change in its position, and different slump values of concrete. The more important defect which is related to the concrete quality is due to the invisibility and lack of proper vibration of concrete which might interfere with its quality control and examination. The impossibility of vibration and uniform compaction of concrete are among the basic problems and weaknesses of ordinary concrete in this type of pile. In placing concrete in cast in-situ piles, apart from the abovementioned issues, due to the structural element nature, there is no possible way for full compaction. Therefore, it is necessary to use self-compacting concrete (SCC) in this type of structure. The early research works on SCC were published in Japan in the years 1989 to 1991. These studies were concentrated on new properties of concrete such as filling capacity, yielding, and resistance against separation^1,2^. Sweden was the first European country which started the development of SCC, and in 1993 extensive research started under the project name Brite-Euam through the collaboration of European countries. In this research the concretes were classified according to the combination of Portland cement and limestone powder and implemented for civil and housing projects as experimental projects^3^. In these combined cement, use was made of European concrete committee guidelines^4^. Also, this research started based on ACI 237R^5^ and PCI guidelines^6^. The ACI committee report was published in 2007. Onyelowe et al.^7–11^ used various experiments and Artificial neural networks (ANNs) to comprehensively research the SCC in terms of rheology, thermal characteristics, workability improvement, permeability, and permeation flow, presenting models and recommendations for enhancing the SCC.

Concerning the concrete placing of cast in-situ piles, various research works have been performed. Camp et al.^12^, investigated the methods for concrete placing and preparation which could prevent execution problems during concrete placing of cast in-situ piles. In this research, use was made of high strength concretes with different combinations in cast in-situ concrete piles. Dees & Mullins^13^, investigated the impacts of different parameters on the behavior of special concretes used in the cast in-situ piles and demonstrated that easy execution of self-compacting concrete is related to such parameters as flowability, stability, finishability, consistency, and pumpability. During concrete filling of piles, due to the lack of forms and impossibility of vibration and also a fixed path of concrete filling, using tremie pipe with a long length and rather a small diameter could affect the acceptability of concrete. The other factors such as the invisibility of drilling direction, rather unknown arrangement of rebar grid, unknown thickness of the concrete cover, and lack of knowledge on the spreading of concrete are effective on the desirability of concert placed in piles and presence of uncertainty in this respect. These uncertainties are greatly decreased by using self-compacting concrete. Therefore considering the widespread application of self-compacting concrete and the advantages provided by using this type of concrete especially when used in concrete piles, it is essential to conduct an accurate investigation in terms of preparation and determination of needed compressive strength (CS) for this type of concrete in the projects.

Recently, numerous research works have elaborated on predicting the compressive strength of different concrete types using various soft calculation techniques. Existing algorithms can find the exact optimal solution. Yet, they are inefficient in complex design optimization problems, with their processing time increasing exponentially in proportion to the number of dimensions of the problem. This makes these models susceptible to computational error and limitations in applicability to various environmental conditions. This indicates the necessity of presenting a new model that can address the limitations of existing models at even lower computational errors with easy implementation steps. Although the developments achieved in information technology and the processing power of computers have provided practitioners with a broader spectrum of tools for predicting the compressive strength of various concrete types, the extensiveness of the data for optimization and presentation of a comprehensive model for predicting the compressive strength of concrete is still beyond the capabilities of conventional systems, indicating the need for identifying and introducing new methods and software tools for presenting more efficient models. Thanks to the high accuracy, flexibility, and novelty of gene expression programming (GEP) as a heuristic algorithm, it was considered in the present research. Trying to predict possible outputs, this method establishes a relation between the independent and dependent variables based on existing information in the data without any presumption about the data structure. The primary novelty of the present research lies in presenting a GEP-based model for predicting the compressive strength of the SCC in cast-in-situ concrete piles. Indeed, despite its comprehensiveness, the GEP is yet to be adequately considered as it is relatively new. Accordingly, only a few recent concrete studies have focused on this algorithm. This study presents a new GEP-based model for estimating the compressive strength of SCC in cast-in-situ piles, providing a reliable alternative to costly and time-intensive tests for compressive strength evaluation.

Table 1 presents the details on the application of soft computing techniques for CS prediction, obtained from relevant literature.

**Table 1:** The researchers about a prediction of CS.

No.	Algorithm	Year	References
1	ANN	2024	^14^
2	ML	2024	^15^
3	MEP	2024	^16^
4	BML	2022	^17^
5	GEP	2021	^18^
6	ANN, GA	2021	^19^
7	ANN, bagging and boosting	2021	^20^
8	GEP	2021	^21^
9	SBRS, GEP, ANFIS	2021	^22^
10	GEP and RF	2020	^23^
11	GEP, DT and Bagging	2020	^24^
12	GEP	2020	^25^
13	ANN, GA	2020	^26^
14	GEP	2020	^27^

## Materials and methods

### Experimentation and neural network model for SCC

The optimal mixture design for concrete is obtained by selecting the available materials which make the concrete executable and ensure reaching the expected strength and other characteristics required for hardened concrete by the designer. Some basic principles that should be considered for self-consolidating concrete are as follows:

### Preparation of self-consolidating concrete mixtures

#### Cement

One of the following specifications: ASTM C150^28^, C595^29^, or C1157^30^ must be present in the cement allowed^5^.

#### Silica fume

The stability of SCC mixtures is increased by silica fume. Reducing the water mobility within the concrete matrix leads to an increase in the mixture stability with the ability of silica fumes. The viscosity of SCC decreased at relatively low replacement rates 5% or below plastic. The shape, size, and distribution of cement particles generally determine the rate of replacement required^5^.

#### Selection of aggregate

Due to the plastic concrete’s good passing ability and stability, the coarse aggregate (CA) maximum nominal size should be selected. To obtain the concrete passing ability, the size of CA and the volume of CA is very important. Therefore, to improve the passing ability, a smaller size proposed in ACI 301^31^ may be considered for the maximum nominal size of CA. In terms of the effect on SCC workability, the CA particle shape is also very important. A rounded CA will impart greater filling ability for the same water content of a mixture than a similar crushed stone. Mixing different stone sizes can be used to improve the overall properties of the mix. If the CA is larger than 12.5 mm, as a guide to minimize SCC blockage through the reinforcement. For congested formwork, the maximum nominal size should then be the absolute CA volume in the concrete volume’s 28 to 32% range. The total CA volume percentage may be higher for applications without aggregate blocking or passing ability concerns^5^.

##### Fine aggregate

A good grade of concrete sand is essential for the fine-aggregate component. To improve the properties of SCC plastic, a combination of natural and manufactured sand may be useful^5^.

##### Coarse aggregate

This CA size range is used in highly congested steel reinforcement or challenging concreting conditions. For the first trial batch, in terms of starting point, an initial ratio of 50% sand and 50% CA (10 mm) will be suitable^5^.

### Compressive strength test on SCC

**Fig. 1:**
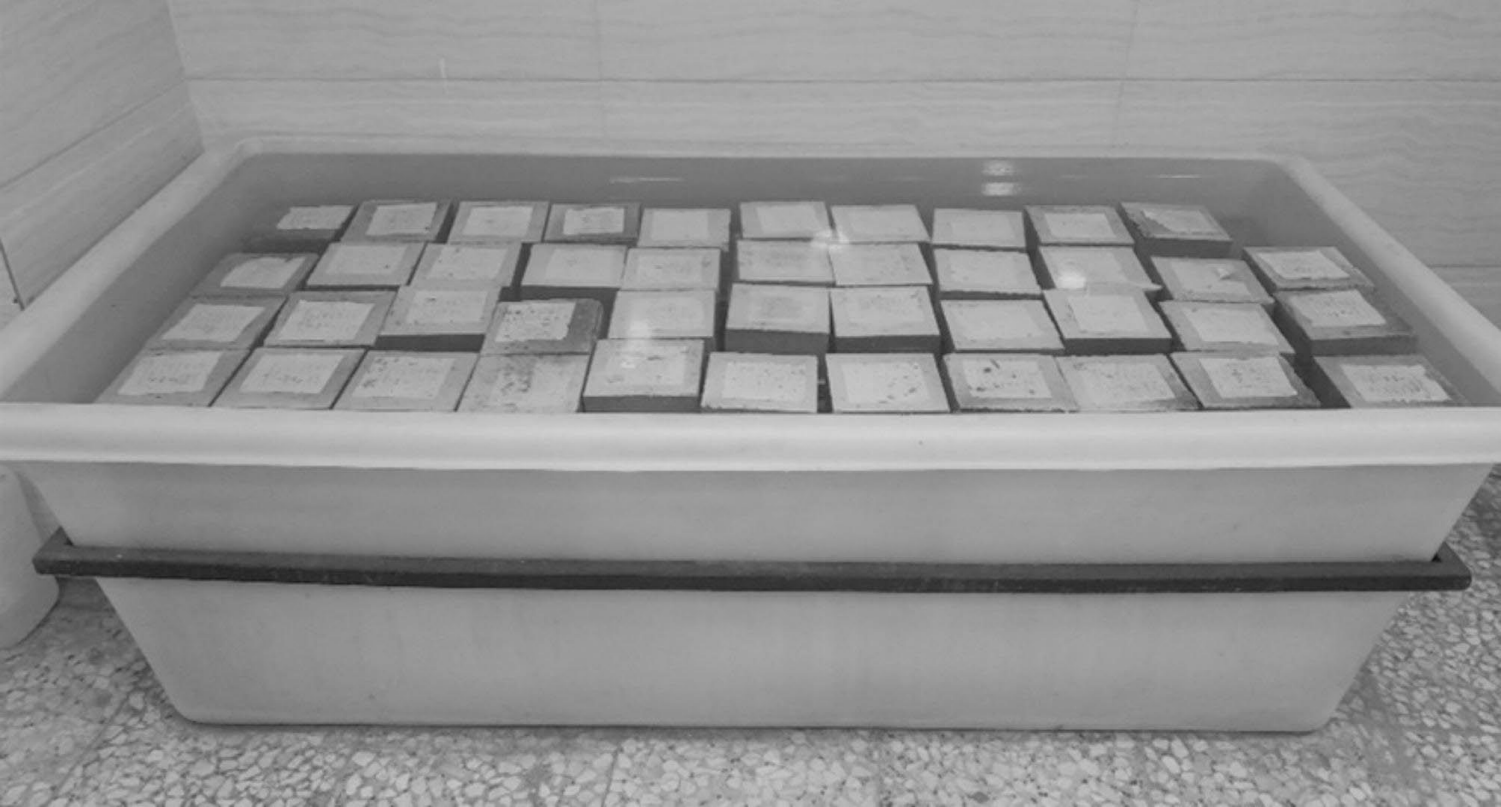
Concrete curing tank.

In this study, the forty-nine 15 × 15 × 15-cm cubic samples of mixture were cast for the compressive strength test and were tested 28 days after casting (Fig. 1).

### Preparing model for self-consolidating concrete in cast in-situ piles mixtures

In this study for predicting the CS degree of self-compacting concrete in cast in-situ piles, a total number of 6 inputs including silica fume, FA, CA, cement, water, and high-reactivity metakaolin were selected. Based on the guidelines from previous research^32^, 70% of the data is assumed for training, 15% for testing, and 15% for validation. Then, using Gene Expression Programming (GEP), a model was presented to estimate the CS of self-compacting concrete in cast in-situ concrete piles. To verify the proper performance of the finally developed model by GEP, a statistical analysis was conducted to evaluate the accuracy of the results. Then, Genetic algorithm (GA) and Artificial neural network (ANN) were further utilized to solve the problem, and their results were compared to those of the presented model.

#### ANNs

Artificial neural networks (ANNs) refer to a branch of machine learning (ML) where principles of neural organization in living organisms are implemented. An ANN is formed by a set of linked nodes called artificial neurons. Taking an artificial intelligence (AI) model as the brain, the neural synapses and processing nodes that follow the information analysis are called AI neural networks. In its simplest form, an ANN is made up of three layers. The first layer is the input layer, in which the data is introduced from the environment in which the AI develops. The input data is processed by input nodes and classified before being dispatched to the second layer. The second layer of an ANN is called the hidden layer, where the outputs from the previous layer are decomposed and processed. Remember that an ANN can have tens of hidden layers. Finally, the output layer encompasses all pieces of data processed by the network and can return the response to the environment. This layer can be made from singular or agglomerated nodes^33^.

#### GA

Genetic algorithm (GA) is a powerful method of solving finite and infinite optimization algorithms based on natural selection phenomenon. It has been further applied in AI optimization algorithms. In fact, the GA is based on Darwin’s theory of evolution and genetics. Based on natural evolution and first developed by John Holland^34^ during the 1960s, this algorithm is usually used to solve problems through optimization. First, the GA randomly generates a predefined number of possible solutions to the problem—each solution is known as a chromosome. The set of all generated solutions is known as a population. Once finished with generating the random population of solutions, the algorithm evaluates all chromosomes based on a predefined fitness function. GAs are especially applied in the optimization of model parameters in NL. This algorithm has been further used to optimize hyper parameters like learning rate, regularization parameters, and network architecture in neural networks.

#### GEP

Gene expression programming (GEP) was first introduced by Ferreira in 1999. As a special GA, it selects individuals from the population based on a fitness criterion and subjects them to one or more genetic operators. In GEP, individuals are encoded as annotated sequences of fixed length (i.e., chromosome) and nonlinearly expressed as trees of various forms and sizes. The structure of each gene is controlled by its head and tail, and this structural form of the genes enables the GEP to establish a valid program for which the chromosome modification limit is not important. GEP starts by generating an initial generation of chromosomes by randomly combining the terminals and functions. A fitness function is then used to evaluate the generation’s significant individuals (i.e., genes). A proper number of individuals are then probabilistically selected from the generation. The probabilistic selection criterion is the ratio of the probability of selecting a better-fitted individual to that of a worse-fitted individual. However, this does not guarantee the non-selection of the worse-fitted individual. When mathematical operators (resembling RNA) and terminals (resembling proteins or chromosomes) perform together, evolutionary emulation becomes possible. At each iteration, a new generation is developed by applying the genetic operators of reproduction, crossover, mutation, and/or replication to a predefined number of selected individuals, followed by evaluating the new generation of individuals using the fitness function^35^.

In this article, GeneXproTools 5.0 was utilized to investigate the accuracy of some experimental data and present a model for estimating the CS of SSC. Table 2 shows the variation ranges of the variables used in this study.

**Table 2:** The variation ranges of the variables.

Variable	Range
High-reactivity metakaolin (d_0_)	2.1–6.6 (kg/m^3^)
Silica fume (d_1_)	24.5–70.7 (kg/m^3^)
Water (d_2_)	166–189 (lit)
Cement (d_3_)	350–505 (kg/m^3^)
CA (d_4_)	738–847 (kg/m^3^)
FA (d_5_)	321–475 (kg/m^3^)

Table 3 shows the GEP configuration that was used for CS of SCC simulation.

**Table 3:** Configuration settings.

	Parameter	Description
General	Fitness function	RMSE
	Number of chromosomes	30
	Genes	3
	Head size	8
	Tail size	9
	Gene size	26
	Linking function	Addition
	Function set	+, -, /, ×, Max2, Avg2, NOT, Ln, 3Rt
Genetic operators	Mutation rate	0.00138
	IS transposition rate	0.00546
	RIS transposition rate	0.00546
	Inversion rate	0.00546
	Gene recombination rate	0.00277
	Gene transposition	0.00277
Numerical constants	Constants per gene	10
	Data type	Floating-point
	Lower bound	− 10
	Upper bound	+ 10

**Fig. 2:**
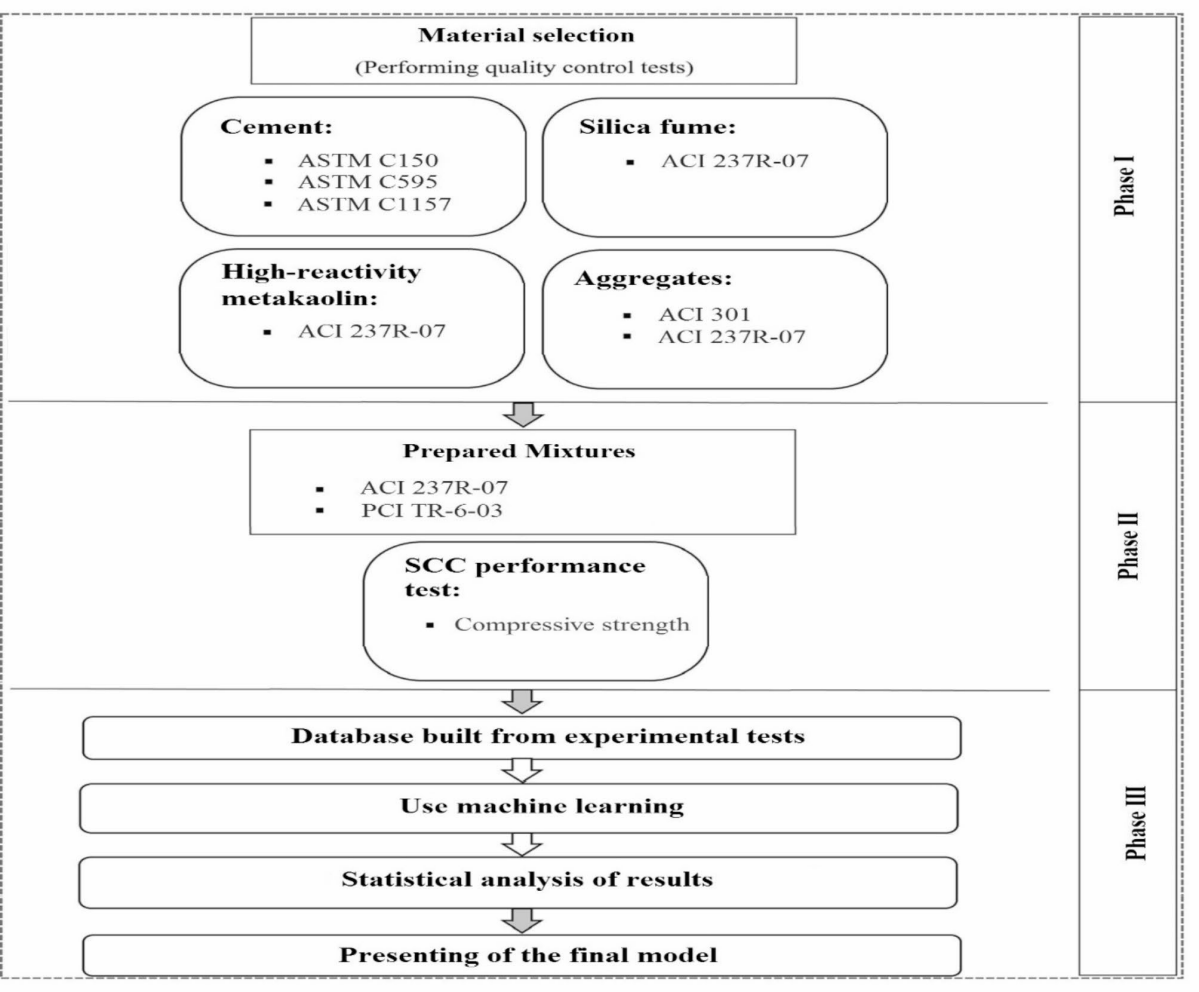
Flowchart of performing this research.

Figure 2 presents the flowchart for performing this research.

The data are given in Table 4.

**Table 4:** Data set of GEP model for estimating CS of SCC.

S No.	Inputs for model						CS of SCC (Outputs)
	Silica fume (kg/m^3^)	High-reactivity metakaolin (kg/m^3^)	Water (lit)	Cement (kg/m^3^)	Coarse aggregate (CA) (kg/m^3^)	Fine aggregate (FA) (kg/m^3^)	Experimentation
1	24.5	2.1	171	350	738	881	321
2	24.7	2.1	169	353	741	882	325
3	25.0	2.1	168	357	745	884	329
4	25.3	2.2	166	362	747	885	331
5	25.7	2.2	168	367	750	885	336
6	25.8	2.2	169	369	753	886	337
7	26.0	2.2	169	372	756	886	339
8	26.3	2.3	168	376	759	887	341
9	26.5	2.3	168	379	762	888	343
10	26.7	2.3	168	381	765	888	345
11	27.0	2.3	169	386	768	889	349
12	27.2	2.3	170	388	769	890	349
13	27.3	2.3	171	390	771	890	353
14	27.5	2.4	172	393	773	891	354
15	27.7	2.4	173	395	775	892	355
16	43.6	4.0	176	396	776	893	356
17	43.7	4.0	178	397	777	893	358
18	43.8	4.0	179	398	778	893	359
19	43.9	4.0	178	399	778	893	361
20	44.1	4.0	176	401	779	893	363
21	44.3	4.0	177	403	779	894	367
22	44.6	4.1	178	405	780	894	369
23	44.8	4.1	179	407	781	894	371
24	45.0	4.1	180	409	782	895	374
25	45.4	4.1	181	413	783	895	379
26	45.5	4.1	176	414	784	895	382
27	45.8	4.2	176	416	785	896	386
28	45.9	4.2	177	417	786	896	389
29	46.1	4.2	178	419	788	896	391
30	46.3	4.2	179	421	790	896	393
31	46.6	4.2	176	424	792	897	395
32	46.8	4.3	175	425	793	898	397
33	47.0	4.3	175	427	794	898	398
34	47.2	4.3	176	429	795	899	399
35	47.5	4.3	177	432	797	900	401
36	48.0	4.4	179	436	798	900	403
37	48.3	4.4	180	439	800	901	408
38	48.6	4.4	181	442	802	901	411
39	62.0	5.8	186	443	803	902	412
40	62.9	5.8	186	449	806	903	425
41	63.4	5.9	187	453	809	904	438
42	64.1	6.0	189	458	814	905	441
43	64.7	6.0	188	462	819	907	447
44	65.7	6.1	187	469	822	909	453
45	66.2	6.1	185	473	828	911	458
46	66.6	6.2	184	476	833	912	461
47	67.9	6.3	183	485	838	914	465
48	69.2	6.4	181	494	841	916	471
49	70.7	6.6	180	505	847	917	475

## Result and discussion

**Fig. 3:**
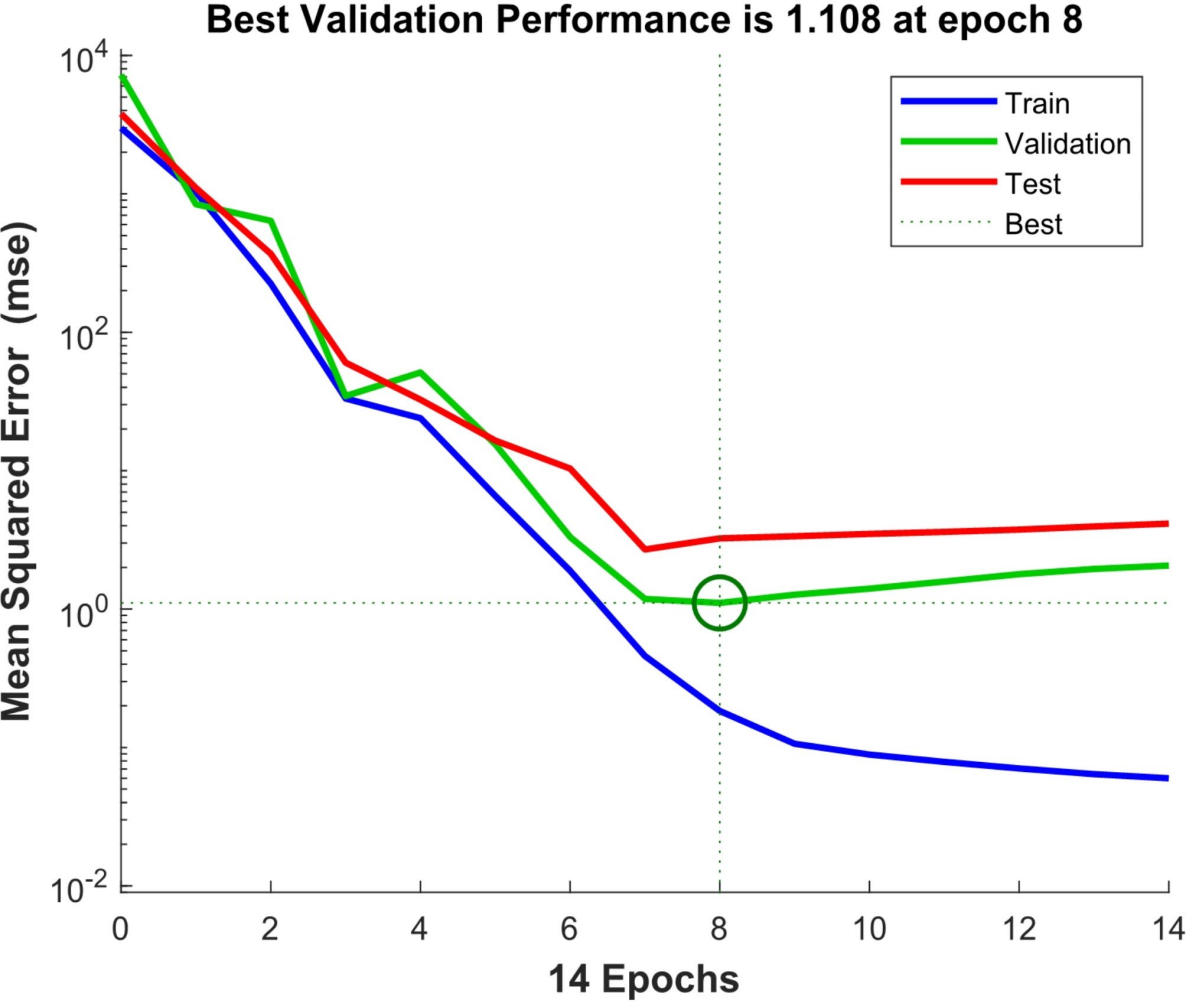
The lowest MSE for the validation set in the ANN.

The criterion used for stopping the training of networks was mean square error (MSE) which is the difference between the mean square of the output and target values. The smaller values indicate the better performance of the network and a zero value means lack of any error. The regression values measure the correlation between outputs and targets in the networks. So that *R* = 1 means full relation and *R* = 0 means a random relation. The MSE and R criteria were selected as the basic criteria for selecting the ideal network. Figure 3 shows the mean square of errors in the network which starts from large values and reduces to smaller values. In other words, this indicates that the network is in the learning state. This diagram has three lines that represent a class of data. Training the vectors continue till the network error in the validation vectors is decreased. After training the network, the learning process is stopped.

**Fig. 4:**
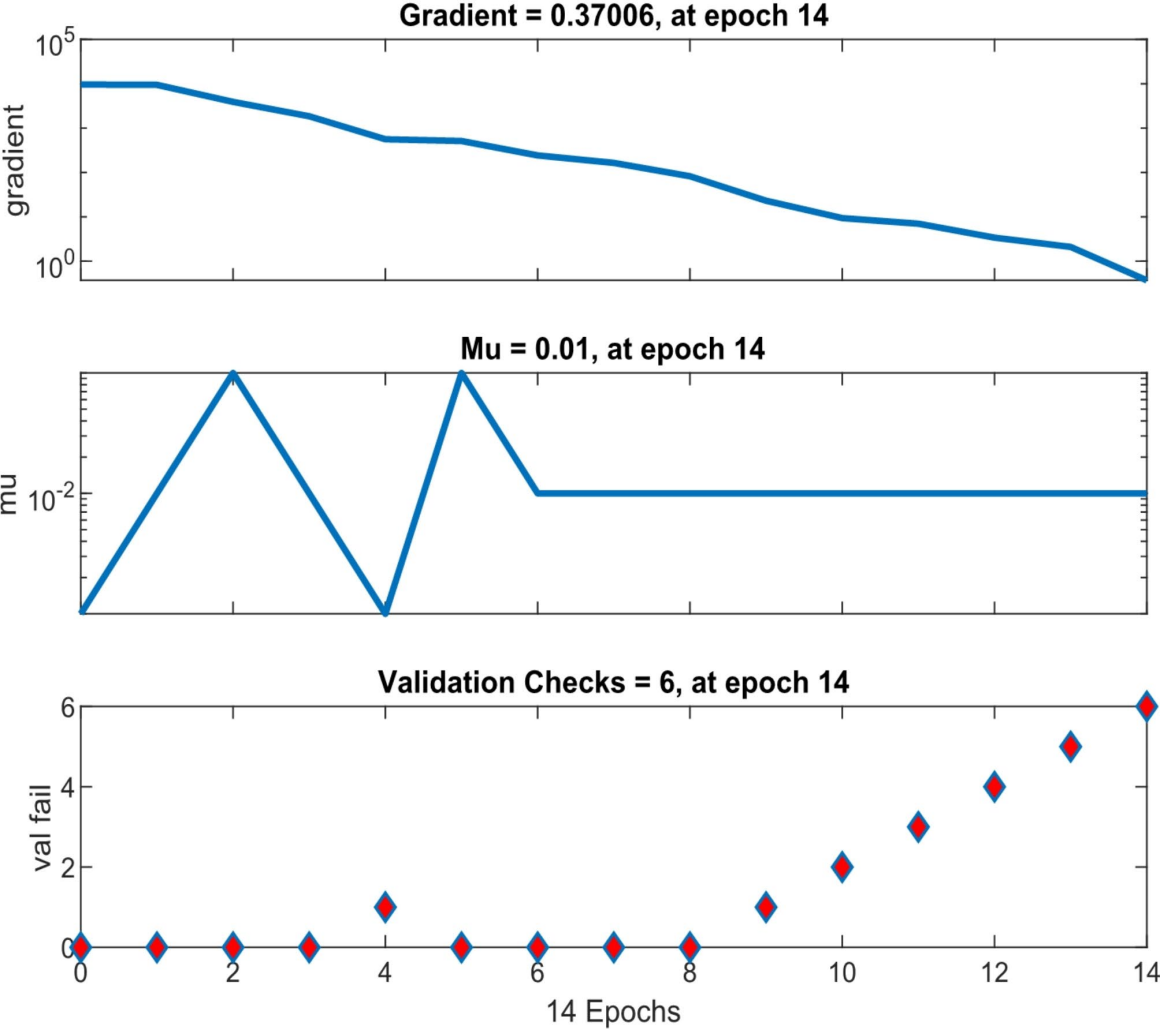
Gradient & validation checks.

The gradient of the mean square error (MSE) and validation studies show that the neural network is convergent. In Fig. 4, the procedure for reaching the MSE obtained from the training process is shown for different data, using the gradient and validation checks. The training process is stopped when the validation checks reach no. 6, based on the default value. The gradient is equal to 0.37006 at epoch 14 and the number of validation checks is 6 at epoch 14.

**Fig. 5:**
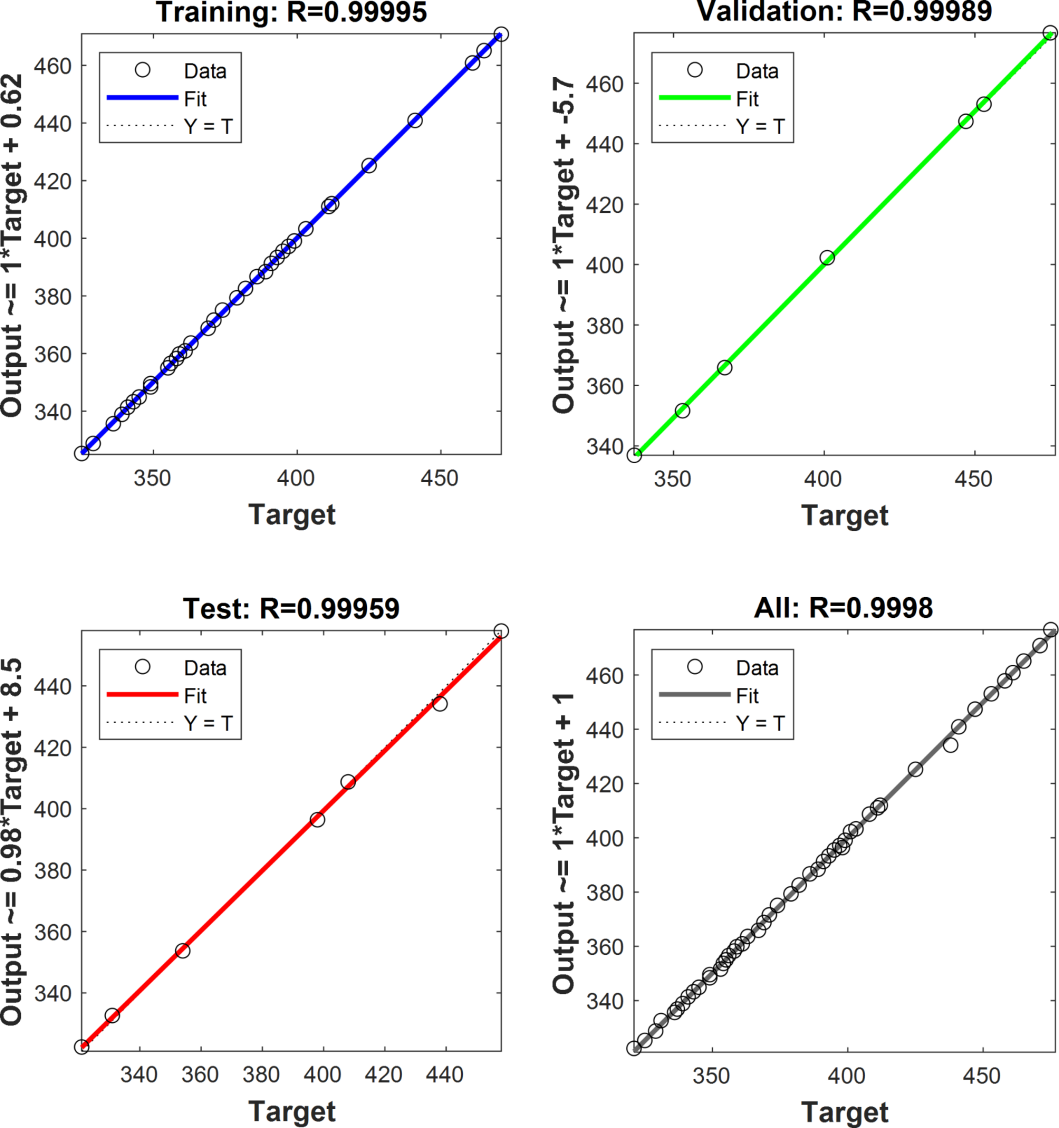
Regression of the data corresponding.

The best network is the one with 10 neurons in its hidden layer, as it has yielded good regression values with minimum mean error among all the networks. The regression for training, validation and training data sets was observed in Fig. 5 which depicts accuracy to of the training to target output data.

**Fig. 6:**
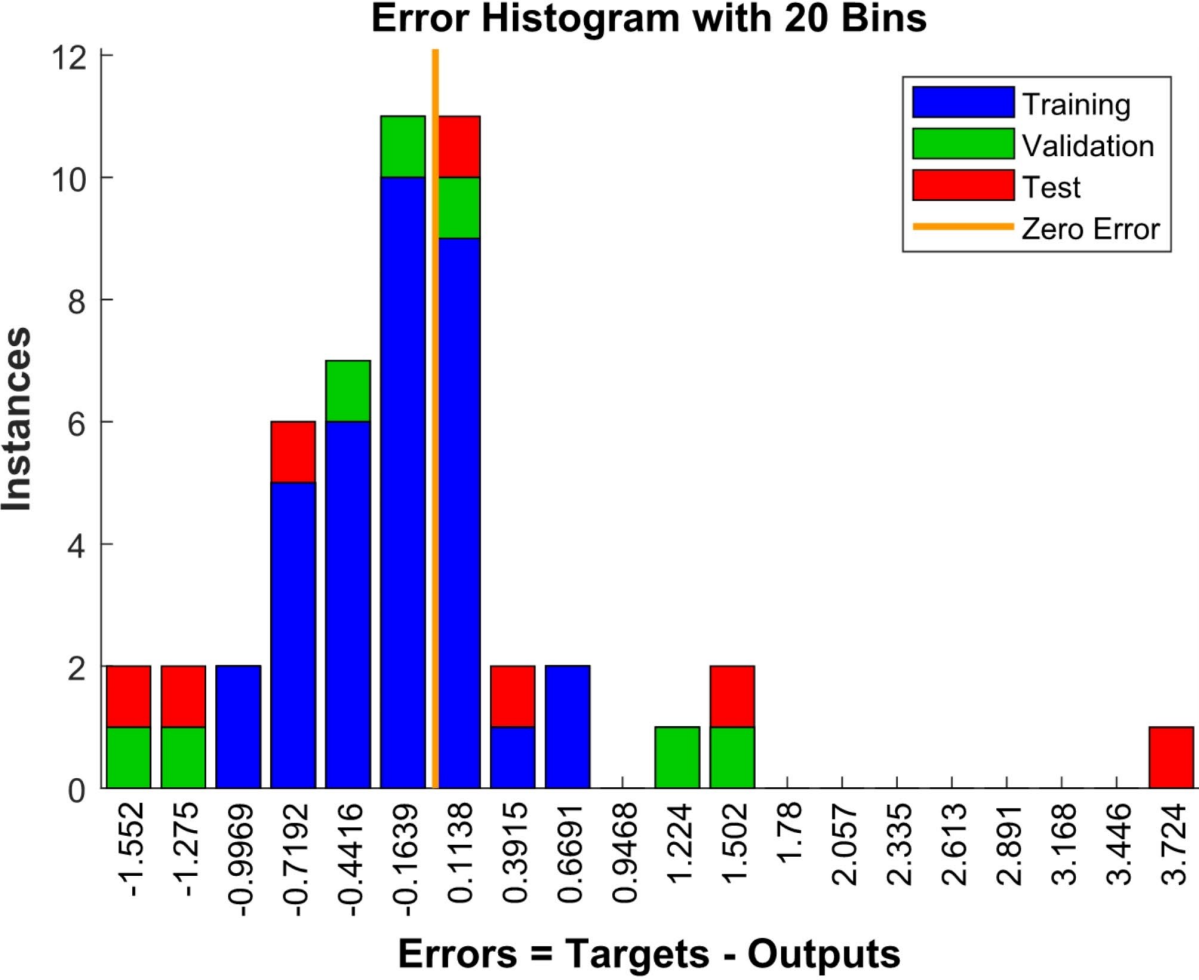
Error histogram.

The error histogram shown in Fig. 6, depicts the artificial neural network performance. The training data are shown in the blue color, the validation data are shown in the green color and the test data are shown in the red color.

**Fig. 7:**
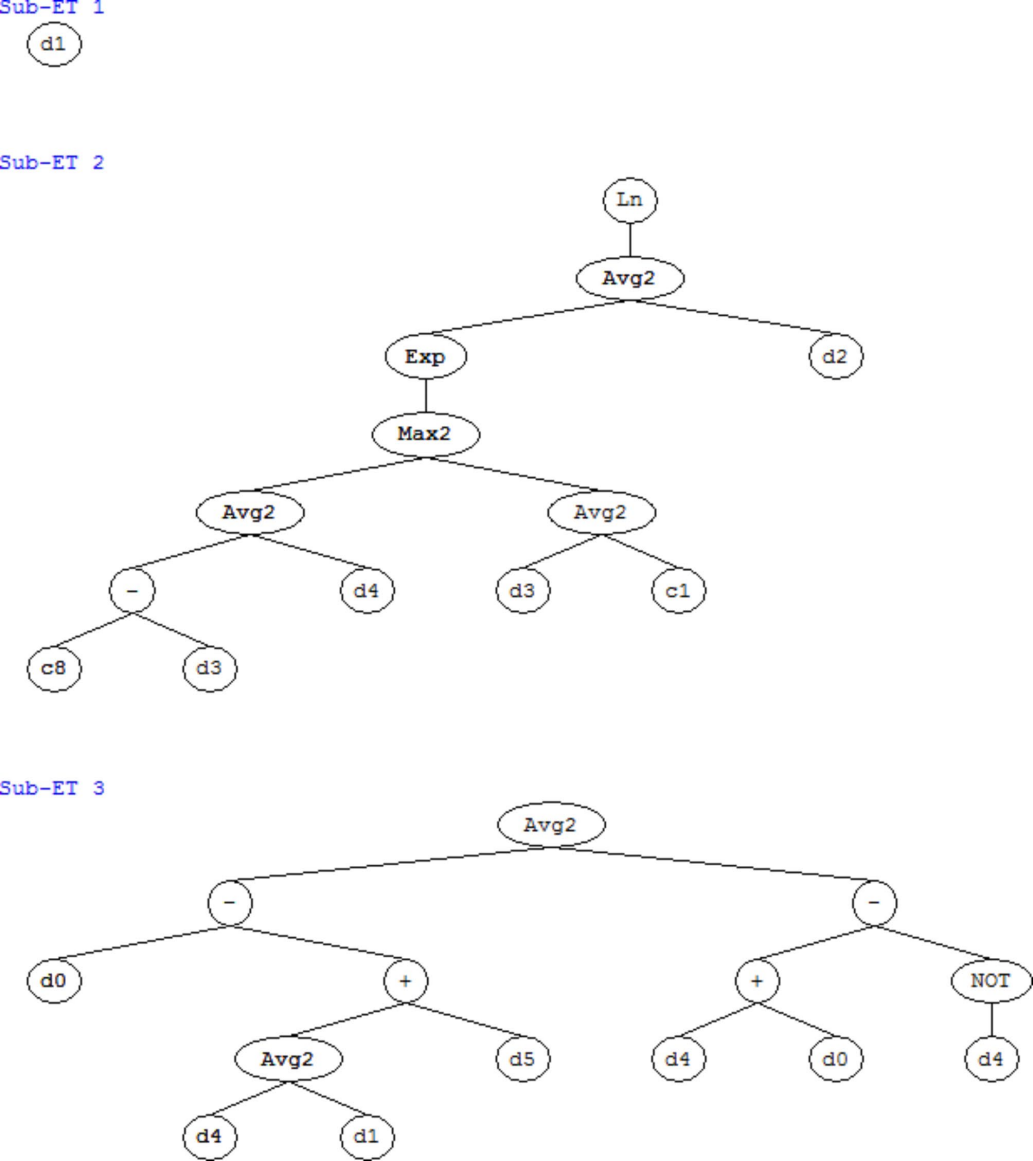
Expression tree of gene expression programing model.

The final expression trees extracted using the gene expression programing is demonstrated in Fig. 7. It consists of three sub-expression trees connected through the addition operator (+). Using Fig. 7, one can obtain a GEP-based equation, for predicting the CS of SCC in cast in-situ piles (CS_SCC_) (Eq. 1).

1 $$\begin{gathered} C{S_{SCC}}=\left\{ {{\text{Silica fume}}} \right\}+\left\{ {\log \left[ {\exp \left( {\frac{{\left( {\hbox{max} \left( {\frac{{\left( {5.2718 - {\text{Cement }}} \right)+\left( {{\text{CA}}} \right)}}{2}} \right),\left( {\frac{{\left( {{\text{Cement }}} \right)+\left( { - 6.6233} \right)}}{2}} \right)} \right)+\left( {{\text{Water}}} \right)}}{2}} \right)} \right]} \right\} \hfill \\ +\left\{ {\frac{{\left( {\left( {{\text{High-reactivity metakaolin}}} \right) - \left( {\left( {\frac{{{\text{CA}}+{\text{Silica fume }}}}{2}} \right)+({\text{FA)}}} \right)} \right) - \left( {\left( {{\text{CA}}+{\text{High-reactivity metakaolin}}} \right) - \left( {1 - {\text{CA}}} \right)} \right)}}{2}} \right\} \hfill \\ \end{gathered}$$

**Fig. 8:**
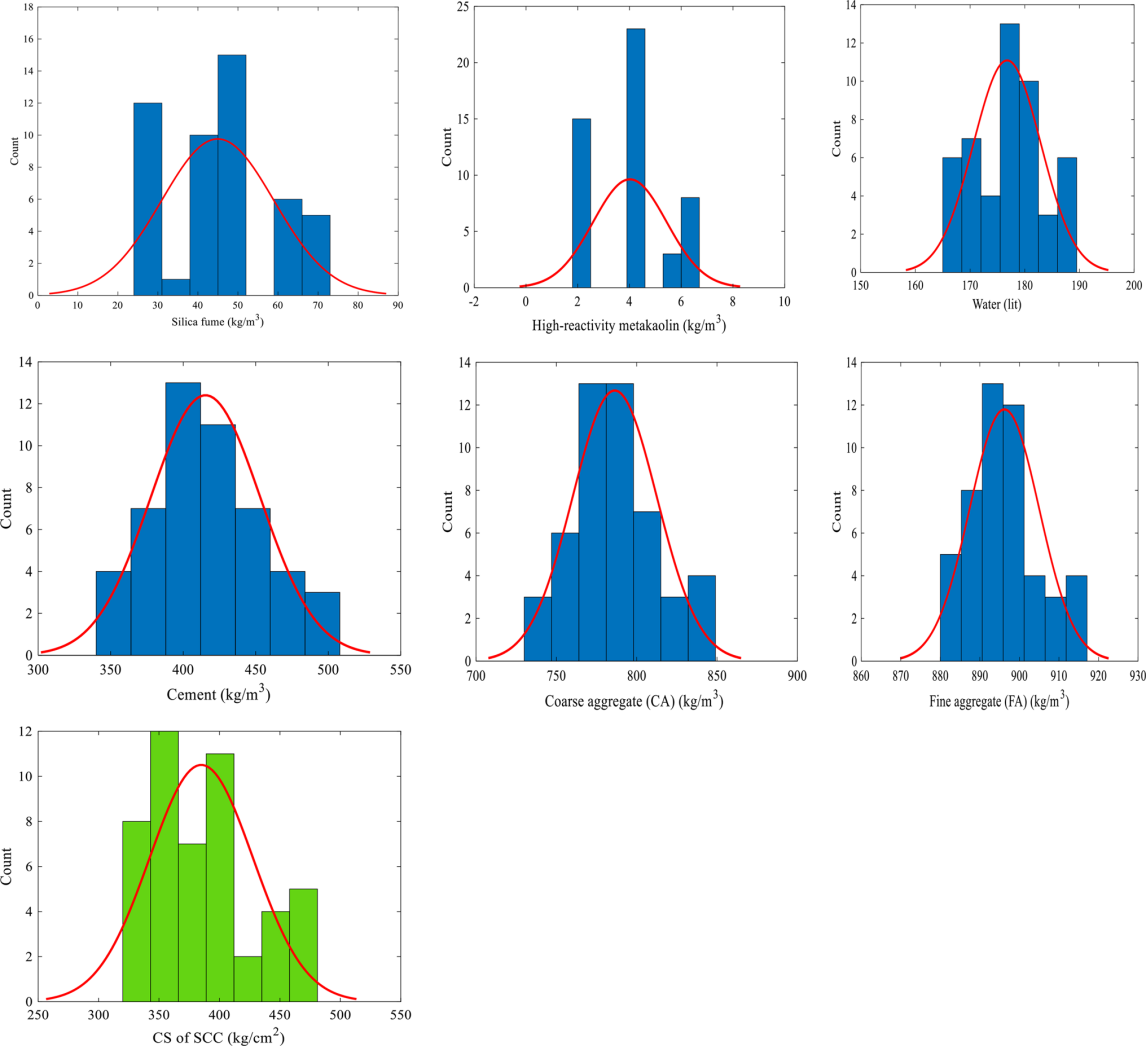
Histograms of inputs (in blue color) and output (in green color) variables.

The statistical parameters summarizing the properties of the variables in the database are presented in Table 5, while Fig. 8 illustrates the distribution of each parameter.

**Table 5:** Statistical measures of variables.

Statistics	Silica fume (kg/m^3^)	High-reactivity metakaolin (kg/m^3^)	Water (lit)	Cement (kg/m^3^)	CA (kg/m^3^)	FA (kg/m^3^)	CS of SCC (kg/cm^2^)
Mean	44.27	4.02	176.78	415.39	786.31	896.20	384.96
Median	45.40	4.10	177.00	413.00	783.00	895.00	379.00
Maximum	70.7	6.60	189.00	505.00	847.00	917.00	475.00
Minimum	24.5	2.10	166.00	350.00	738.00	881.00	321.00
Standard deviation	14.55	1.42	6.17	37.81	26.20	8.78	42.78
Variance	211.78	2.02	38.05	1429.74	686.68	77.12	1830.16

**Fig. 9:**
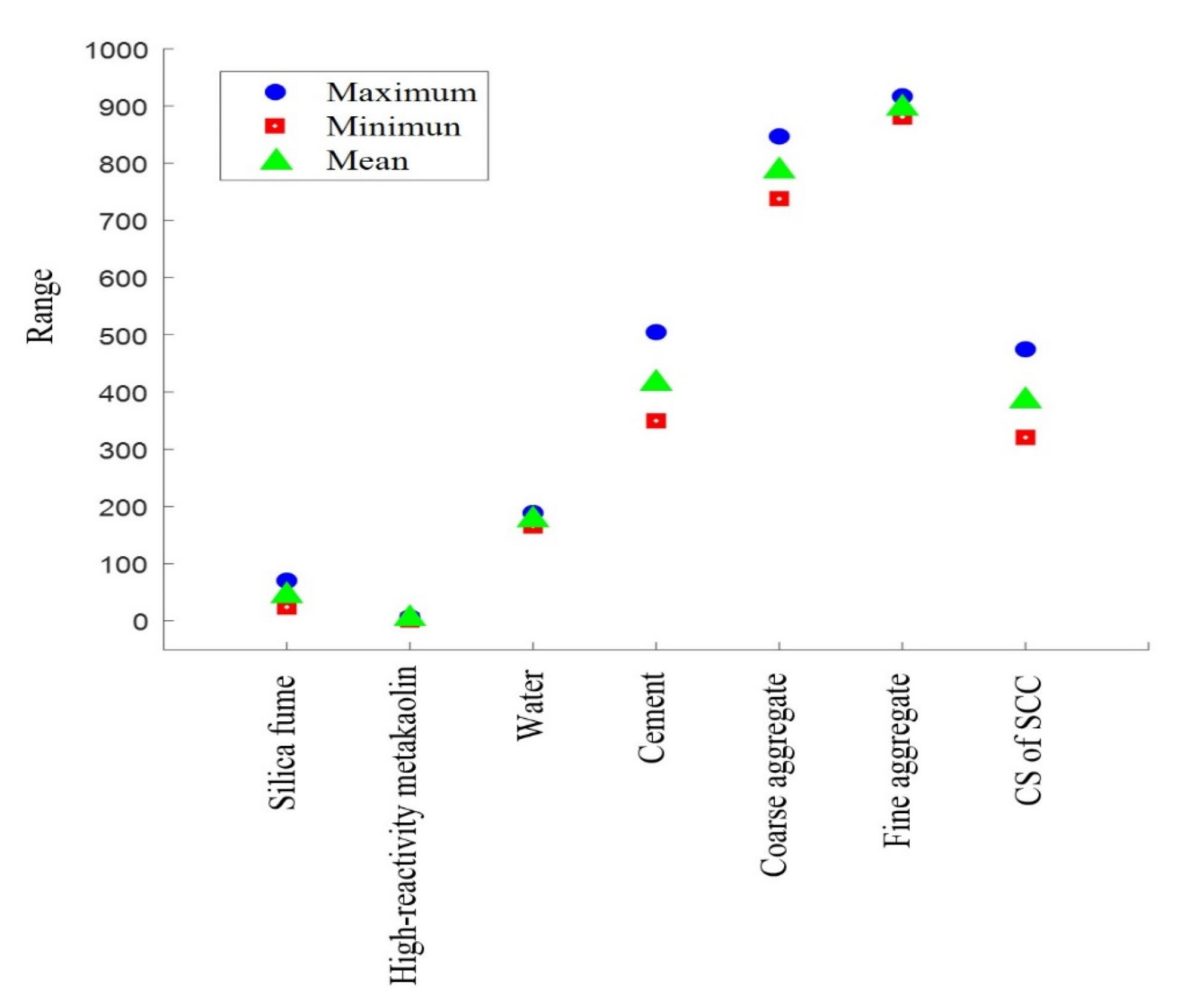
Parameter maximum and minimum value.

In contrast, the maximum and minimum values with their mean are also shown in Fig. 9.

Further, the correlation coefficient was calculated to evaluate the predictive power of the linear relations between the strength of the SCC and the six input parameters, with the results shown in Table 6. The correlation coefficient between any two variables falls in the range of − 1 to + 1. A correlation coefficient of + 1 indicates a perfect and positive correlation, meaning that a unit increase in the value of one variable leads to a unit increase in the value of the other variable. A zero correlation coefficient identifies absolutely unrelated variables, while a correlation coefficient of − 1 indicates a perfect yet negative correlation, meaning that a unit increase in one variable leads to a unit decrease in the value of the other variable.

**Table 6:**
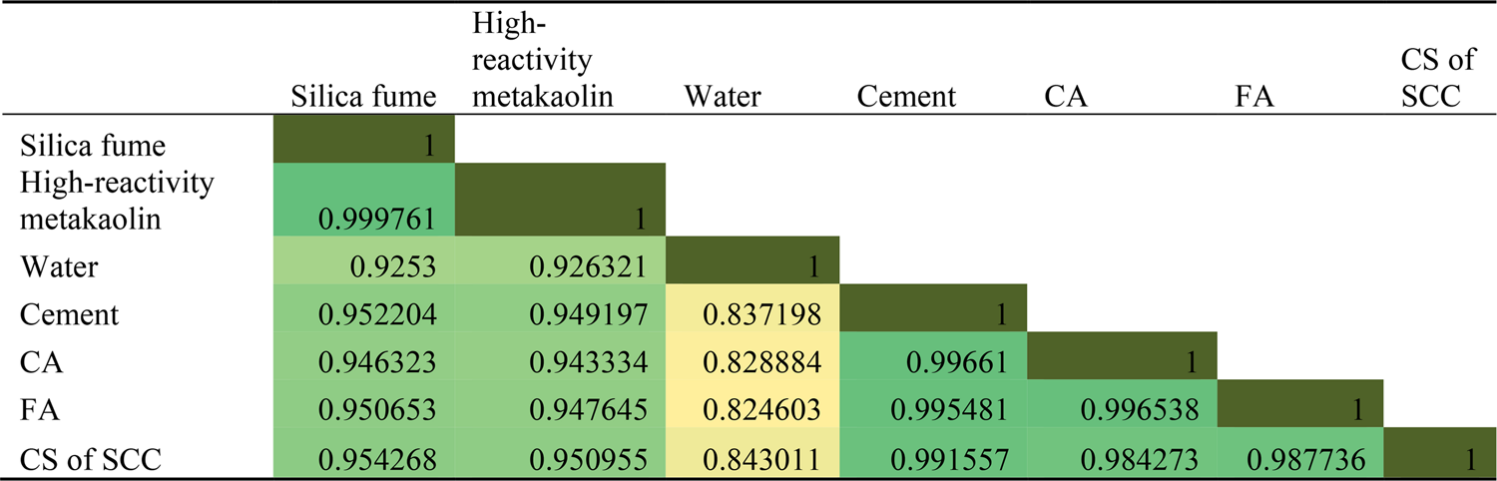
Correlation coefficient results.

Results show that CA and Cement has the largest impacts on the compressive strength test result on the SCC samples, while other input variables exhibit weaker correlations to the CS. Therefore, the selected input variables can be seen as perfect estimators of compressive strength for SCC in cast-in-situ piles.

**Fig. 10:**
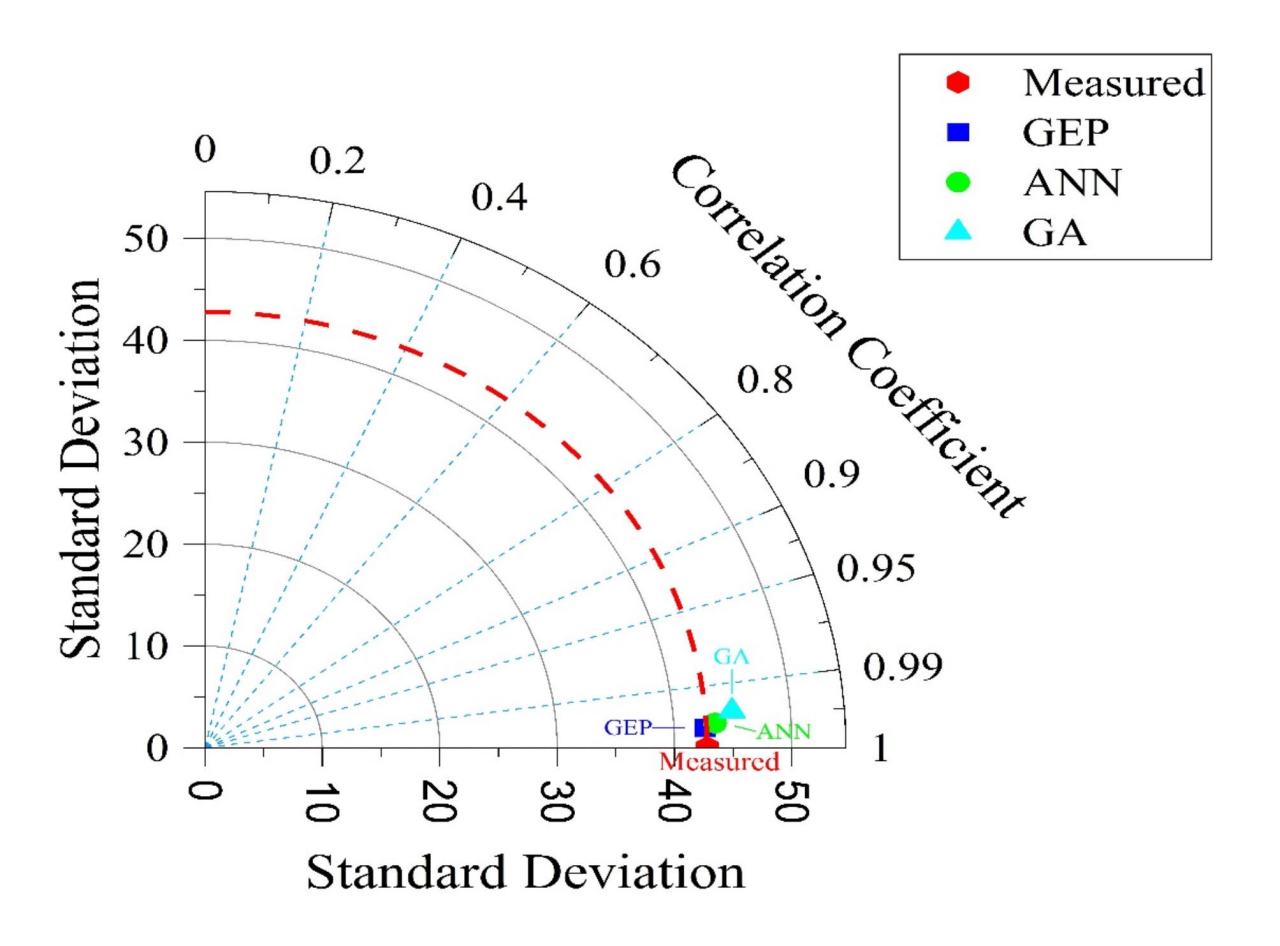
Comparing the accuracies of the developed models using Taylor chart.

For the sake of comparison, the outputs of the three models (i.e., GA, ANN, and GEP) were demonstrated on a Taylor chart (Fig. 10).

A Taylor chart is usually devised to compare multiple modeling outputs in the same diagram, where the levels of error, correlation, and standard deviation of the modeling outputs can be compared^36^.

The outputs of the GEP and ANN models exhibited tiny deviations from one another, so one could reasonably evaluate them as identical. The dashed lines indicating the radii of the quarter circle in the Taylor chart show Pearson’s correlation coefficient (i.e., R). The outputs of the GEP and ANN are very close to the *R* = 1 dashed line, highlighting their excellent accuracy. It can then be stipulated that the GEP model is slightly more robust than the ANN. The outputs of the GA are somewhat closer to the *R* = 0.99 dashed line. Comparison of the standard deviations against observed data proved the proper performance of the three models in estimating the compressive strength of SCC.

## Conclusions

Selection of a proper mix design for making concrete with the CS desired by the designer is of great importance. In this research, the experimental SCC mix designs were successfully modeled using the ANNs. The input variables in the SCC mix designs to predict the CS degree of self-compacting concrete in cast in-situ concrete piles were selected accurately based on the laboratory results. The values of CS of the SCC specimens were used for the training, validation, and testing of the neural network, and the obtained results were desirable. This means proper validation and training of the ANN. The analysis of the simulated model proved this assumption that by extracting the parameters corresponding to the mix design and the experimental results and also the application of the ANNs one could predict the CS of the SCC in cast in-situ concrete piles with a high precision equal to 99.98%.

The results obtained from this research are as follows:

- The best type of concrete in technical and execution terms for cast in-situ concrete piles is self-compacting concrete, provided that a proper balance is established between the flowability and viscosity of concrete.
- Due to increased compressive strength using self-compacting concrete, assuming a fixed section and length for the pile, the percentage of reinforcement at the section is reduced.
- Due to the relatively high compressive strength of self-compacting concretes concerning ordinary concretes with very high slumps, the durability, and impermeability of the used concrete in the pile also increase.
- The application of self-compacting concrete significantly reduces the uncertainties associated with ordinary concrete used in piles.

Limitations of the present research include the procurement of standard materials according to relevant procedures and standard codes. Moreover, preparing experimental specimens of self-compacting concrete is a susceptible process that needs lots of knowledge and experience. Also, the developed model is valid within the considered range of each parameter, beyond these ranges, the model must be verified.Considering the importance of the SCC and its compressive strength, especially regarding the cast-in-situ piles, one can further study other methods, such as adaptive neuro-fuzzy inference system (ANFIS), multi-layer perceptron (MLP), multivariate adaptive regression splines (MARS), etc. and compare their results to identify the best and most efficient method. Confirming the high accuracy of the presented model, results of the present research showed that the proposed GEP algorithm is easy to implement and highly accurate and offers lower levels of estimation error coupled with no limitation in terms of input parameters, justifying its applicability as an alternative to costly and time-intensive experiments that should be otherwise used by engineers, companies, and research institutes to estimate the compressive strength of SCC in cast-in-situ piles.

## Data Availability

The datasets used and/or analysed during the current study available from the corresponding author on reasonable request.
